# Supplementary material for: Early-Stage Corticostriatal Circuit Hyperactivity Impairs Cholinergic Function and Cognitive Flexibility in an Alzheimer’s Model
Source: bioRxiv. 2026 Jan 14:2026.01.13.699380. Preprint. [Version 1] doi: 10.64898/2026.01.13.699380 (PMC12871269; doi:10.64898/2026.01.13.699380)
Supplement: Supplement 1 [file NIHPP2026.01.13.699380v1-supplement-1.pdf]

bioRxiv preprint doi: https://doi.org/10.64898/2026.01.13.699380; this version posted January 14, 2026. The copyright holder for this preprint (which was not certified by peer review) is the author/funder, who has granted bioRxiv a license to display the preprint in perpetuity. It is made available under aCC-BY 4.0 International license.

## Supplementary Figures

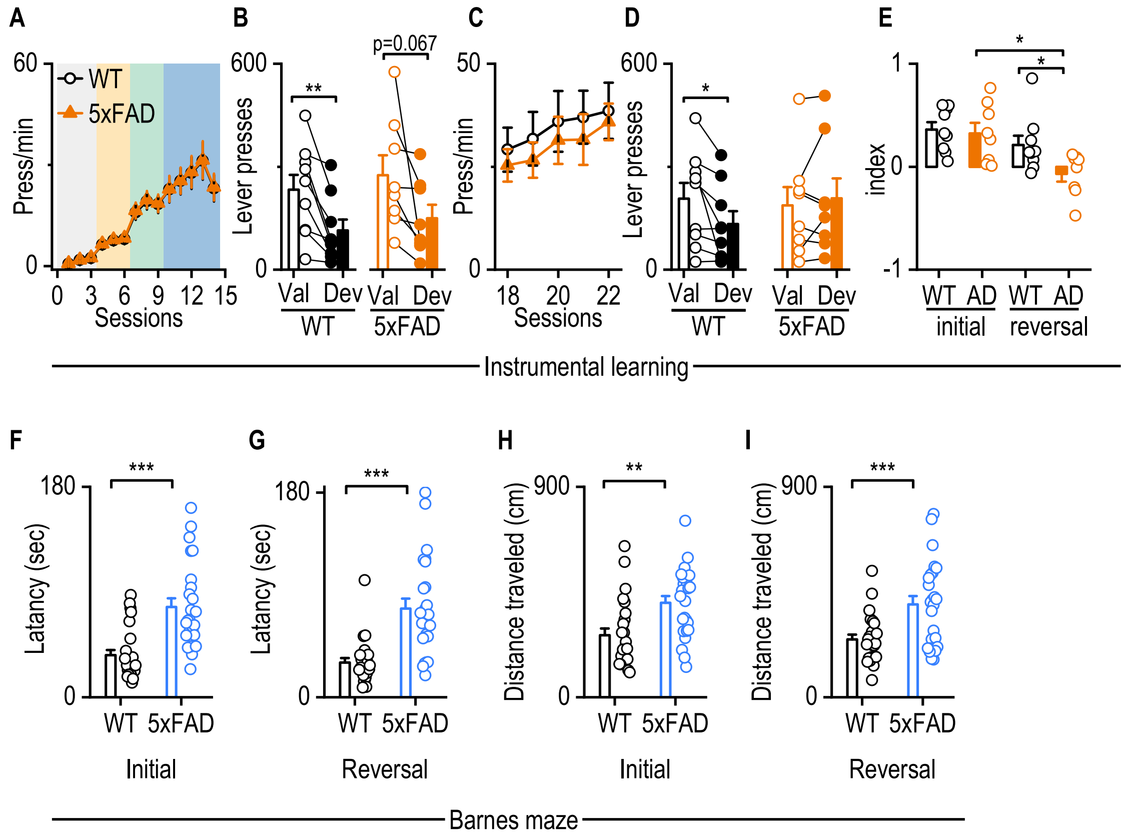


**Supplementary Figure 1. 5xFAD mice exhibited reversal learning deficits in instrumental learning and Barnes maze test at 6 months of age.**

**A,** 6-month-old WT and 5xFAD mice did not differ in lever pressing rates during the initial contingency training. Two-way RM ANOVA with Geisser-Greenhouse correction. $F_{(1,15)} = 0.266$, $p = 0.614$. n = 9 (WT); 8 (5xFAD).

**B,** WT mice displayed fewer lever presses for devalued than valued outcomes during the initial devaluation test, and 5xFAD mice exhibited a similar trend. Paired t-test, $t_8 = 4.059$, p = 0.0036 for WT; $t_8 = 2.169$, p = 0.067 for AD. **$p < 0.01$. n = 9 (WT); 8 (5xFAD).

**C,** WT and 5xFAD animals had similar lever pressing rates during reversed contingency training. Two-way RM ANOVA with Geisser-Greenhouse correction, $F_{(1,15)} = 0.305$, $p = 0.589$. n = 9 (WT); 8 (5xFAD).

bioRxiv preprint doi: https://doi.org/10.64898/2026.01.13.699380; this version posted January 14, 2026. The copyright holder for this preprint (which was not certified by peer review) is the author/funder, who has granted bioRxiv a license to display the preprint in perpetuity. It is made available under aCC-BY 4.0 International license.

**D**, WT, but not 5xFAD, mice displayed fewer lever presses for devalued than valued outcomes during the reversal devaluation test. Paired t-test, $t_8 = 2.569$, p = 0.0332 for WT; $t_7 = 0.843$, p = 0.427 for AD. *$p < 0.05$. n = 9 (WT); 8 (5xFAD).

**E,** 5xFAD mice displayed a significantly lower reversal index than their initial devaluation index and the reversal devaluation index of WT mice. Two-way RM ANOVA with Geisser-Greenhouse correction, $F_{(1,15)} = 4.504$, p = 0.051; Sidak's multiple comparisons post-hoc analysis, *p < 0.05 for sessions within AD. *$p < 0.05$. n = 9 (WT); 8 (5xFAD).

**F and G,** 6-month-old 5xFAD mice spent more time finding the escape box than WT mice did in both initial (F, U = 90, $p < 0.001$) and reversal sessions (G, U = 78, $p < 0.001$). Mann Whitney test, ***$p < 0.001$. n =25 WT and 5xFAD.

**H and I,** The total traveled distance from the starting point to the escape box by 6-month-old 5xFAD mice was higher than that of WT mice in both initial (H, $U = 144$, $p < 0.01$) and reversal sessions (I, $t_{48} = -3.538$, $p < 0.001$). Mann Whitney test or unpaired t-test, ** $p < 0.01$, ***$p < 0.001$. n =25 WT and 5xFAD.

bioRxiv preprint doi: https://doi.org/10.64898/2026.01.13.699380; this version posted January 14, 2026. The copyright holder for this preprint (which was not certified by peer review) is the author/funder, who has granted bioRxiv a license to display the preprint in perpetuity. It is made available under aCC-BY 4.0 International license.

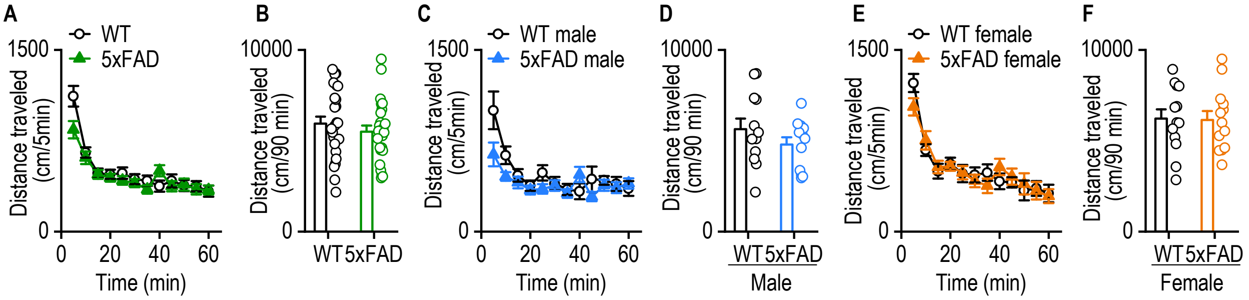


**Supplementary Figure 2. 5xFAD mice did not exhibit abnormal locomotor activity.**

**A**, **B**, Similar spontaneous locomotor activities were shown in WT and 5xFAD mice in time-course (A, $F_{(1, 48)}$ = 0.7615, $p$ = 0.3872) or the total traveled distance (B, $t_{48}$ = 0.873, $p$ > 0.05). Two-way RM ANOVA and unpaired t-test. n = 25 WT and 5xFAD.

**C-F**, There were no differences in spontaneous locomotor activities between males (C, $F_{(1, 22)}$ = 1.466, $p$ = 0.2388; D, $t_{22}$ = 1.211, $p$ > 0.05) or females (E, $F_{(1, 24)}$ = 0.009951, $p$ = 0.9214; F, $t_{24}$ = 0.0988, $p$ > 0.05) 5xFAD mice and their WT control. Two-way RM ANOVA and unpaired t-test. n = 12 male WT and AD; 13 female WT and 5xFAD.

bioRxiv preprint doi: https://doi.org/10.64898/2026.01.13.699380; this version posted January 14, 2026. The copyright holder for this preprint (which was not certified by peer review) is the author/funder, who has granted bioRxiv a license to display the preprint in perpetuity. It is made available under aCC-BY 4.0 International license.

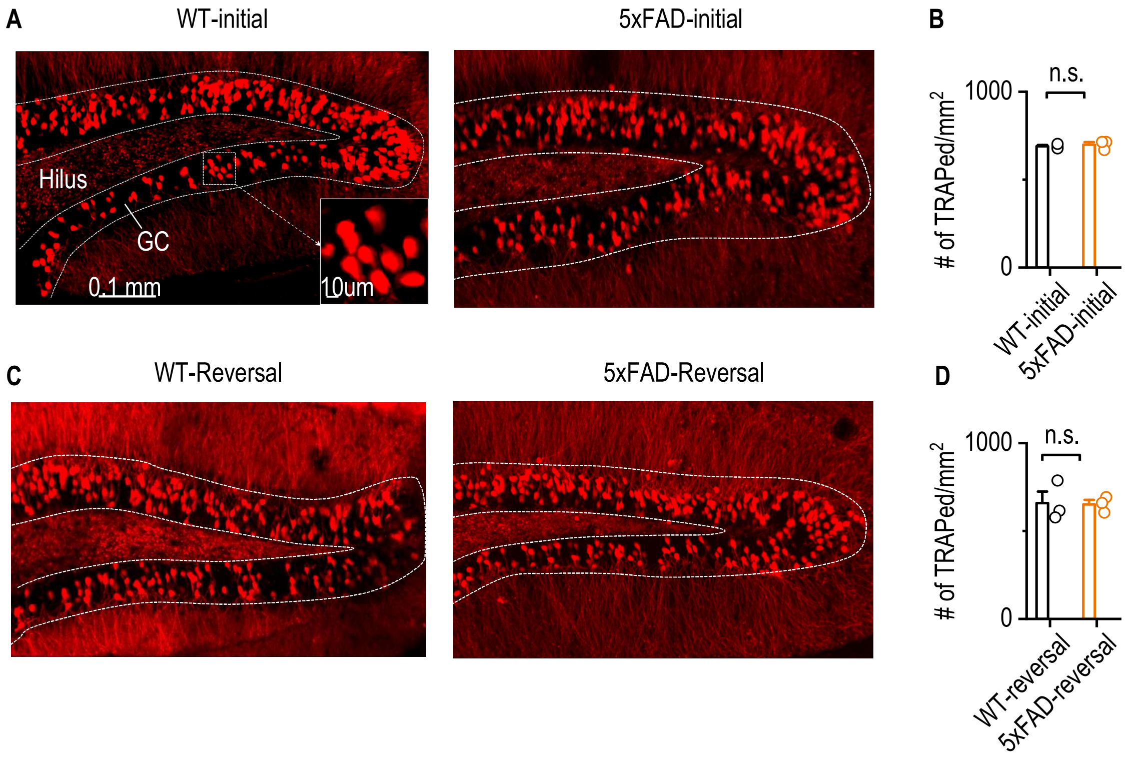


**Supplementary Figure 3. Trapped hippocampus neurons do not differ between WT and 5xFAD in either initial or reversal learning.**

**A,** Representative images showing trapped neurons in the dentate gyrus (DG) of the hippocampus following initial learning in WT and 5xFAD mice. GC, granule cell layer.

**B,** Quantification of tdTomato-labeled DG neurons during initial learning revealed no significant difference between WT and 5xFADmice. $t_4 = 0.5449$, $p = 0.6148$, unpaired t-test. n = 3 mice for both WT and 5xFAD.

**C,** Representative images showing trapped DG neurons during reversal learning in WT and 5xFAD mice.

**D,** Quantification of labeled DG neurons during reversal learning showed no significant difference between WT and 5xFAD mice. $t_4 = 0.1146$, $p = 0.9143$, unpaired t-test. n = 3 mice for both WT and 5xFAD.

bioRxiv preprint doi: https://doi.org/10.64898/2026.01.13.699380; this version posted January 14, 2026. The copyright holder for this preprint (which was not certified by peer review) is the author/funder, who has granted bioRxiv a license to display the preprint in perpetuity. It is made available under aCC-BY 4.0 International license.

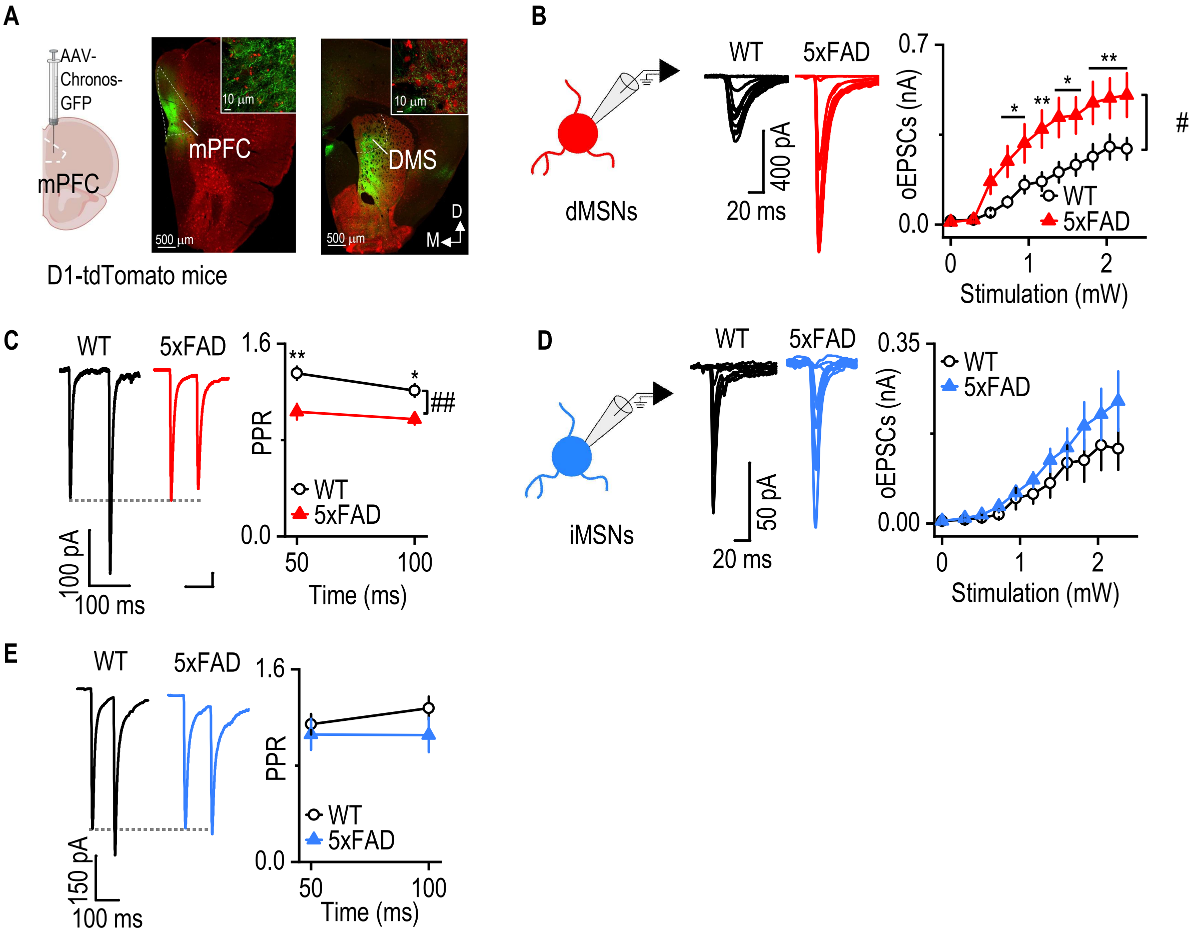


**Supplementary Figure 4. The mPFC-to-dMSN circuit is hyperactive in 12-month-old 5xFAD mice.**

**A,** Representative image showing Chronos-eGFP expression in the injection site (mPFC) and its projection to the DMS in 5xFAD;D1-tdTomato mice.

**B,** oEPSC amplitude in DMS dMSNs was greater in 12-month-old 5xFAD mice than WT controls. Two-way RM ANOVA, $F_{(1,32)} = 5.422$, $p = 0.026$, $^{\#}p < 0.05$; Sidak's multiple comparisons post-hoc analysis, $^{*}p < 0.05$, $^{**}p < 0.01$, versus WT at the same stimulating intensities. N = 17 neurons from 3 mice (WT and 5xFAD).

**C**, PPRs oEPSCs in DMS dMSNs were smaller in 12-month-old 5xFAD mice than age-matched WT controls. Two-way RM ANOVA, $F_{(1,27)} = 9.080$, $p = 0.006$, $^{\#\#}p < 0.01$; Sidak's multiple

bioRxiv preprint doi: https://doi.org/10.64898/2026.01.13.699380; this version posted January 14, 2026. The copyright holder for this preprint (which was not certified by peer review) is the author/funder, who has granted bioRxiv a license to display the preprint in perpetuity. It is made available under aCC-BY 4.0 International license.

comparisons post-hoc analysis, ***p* $< 0.01$, versus WT at the same time interval. n = 15 neurons from 3 mice (WT) and 14 neurons from 3 mice (5xFAD).

**D,** oEPSC amplitudes in DMS iMSNs were not significantly different between 12-month-old 5xFAD and WT mice. Two-way RM ANOVA, $F_{(1, 19)} = 1.167$, $p = 0.2935$. n = 9 neurons from 2 mice (WT) and 12 neurons from 3 mice (5xFAD).

**E**, PPRs of oEPSCs in iMSNs did not differ between 5xFAD and WT mice. Two-way RM ANOVA, $F_{(1, 14)} = 0.07133$, $p = 0.7933$. N = 6 neurons from 2 mice (WT) and 10 neurons from 3 mice (5xFAD).

bioRxiv preprint doi: https://doi.org/10.64898/2026.01.13.699380; this version posted January 14, 2026. The copyright holder for this preprint (which was not certified by peer review) is the author/funder, who has granted bioRxiv a license to display the preprint in perpetuity. It is made available under aCC-BY 4.0 International license.

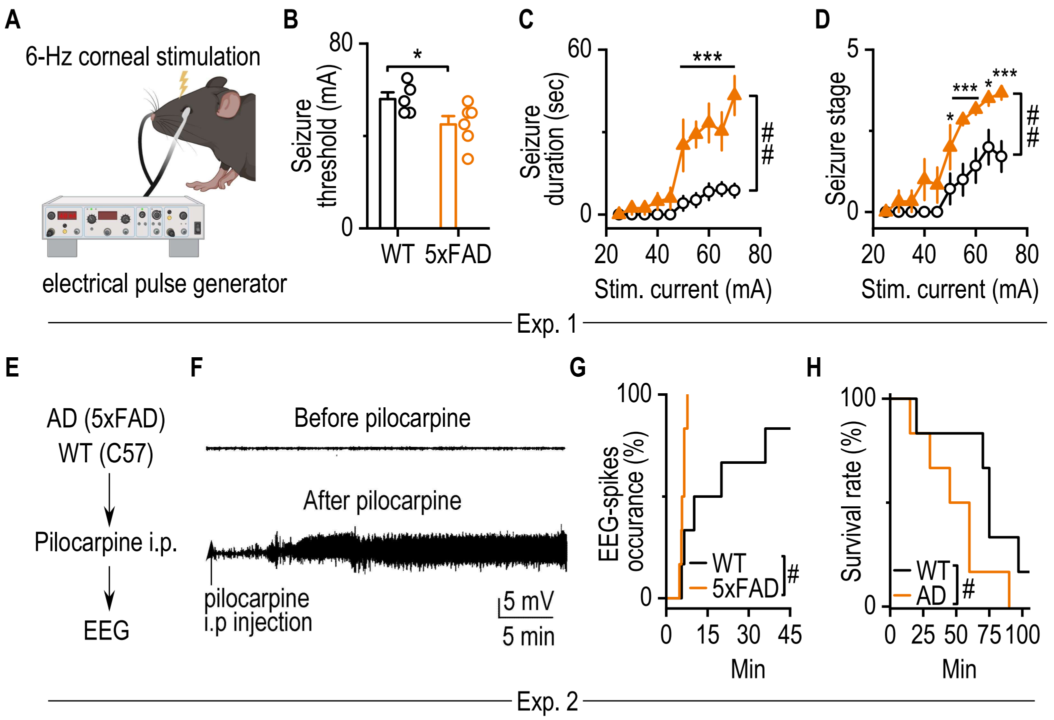


**Supplementary Figure 5. 5xFAD mice exhibit heightened induced seizure activity.**

**A,** Schematic illustrating 6-Hz corneal stimulation in mice. Corneal stimulation consisted of monopolar rectangular pulses (0.2-millisecond duration) delivered at 6 Hz for 3 sec using a constant-current device.

**B,** 5xFAD mice displayed a significantly lower threshold for seizure induction compared to WT controls. $^{*}p < 0.05$, unpaired t-test. n = 6 (5xFAD) and 7 (WT) mice.

**C,** 5xFAD mice had significantly longer seizure durations than WT controls. $^{\#\#}p < 0.01$; $^{***}p < 0.001$ versus WT at the same stimulating intensities, two-way RM ANOVA. n = 6 (5xFAD) and 7 (WT) mice.

**D,** 5xFAD mice exhibited increased seizure stages compared to WT controls. $^{\#\#}p < 0.01$; $^{*}p < 0.05$, $^{***}p < 0.001$ versus WT at the same stimulating intensities two-way RM ANOVA. n = 6 (5xFAD) and 7 (WT) mice.

bioRxiv preprint doi: https://doi.org/10.64898/2026.01.13.699380; this version posted January 14, 2026. The copyright holder for this preprint (which was not certified by peer review) is the author/funder, who has granted bioRxiv a license to display the preprint in perpetuity. It is made available under aCC-BY 4.0 International license.

**E,** Schematic of the experimental design for an additional cohort of AD and WT mice in Exp. 3. Pilocarpine was administered intraperitoneally to induce seizure behavior, and EEG spikes were recorded from the cortex.

**F,** Sample trace in EEG recordings showing spikes before and after pilocarpine i.p. injection.

**G,** 5xFAD mice displayed a higher percentage of seizure spike occurrences in EEG recordings within 45 min after pilocarpine injection compared to WT controls. $^{\#}p < 0.05$, Kaplan-Meier estimates followed by the Tarone-Ware test. n = 6 mice per group.

**H,** Survival rate following seizure induction was significantly lower in 5xFAD mice compared to WT controls. $^{\#}p < 0.05$, Kaplan-Meier estimates followed by the Tarone-Ware test. n = 6 mice/group.

bioRxiv preprint doi: https://doi.org/10.64898/2026.01.13.699380; this version posted January 14, 2026. The copyright holder for this preprint (which was not certified by peer review) is the author/funder, who has granted bioRxiv a license to display the preprint in perpetuity. It is made available under aCC-BY 4.0 International license.

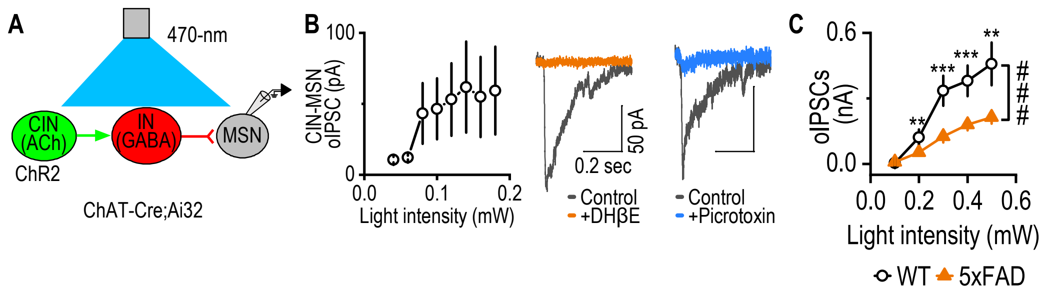


**Supplementary Figure 6. Reduced CIN-to-MSN inhibition in 5xFAD mice.**

**A,** Schematic illustrating the stimulation and recording of CIN-to-MSN inhibitory transmission in DMS slices from 4-month-old ChAT-Cre;Ai32 mice. IN, interneuron.

**B,** Representative CIN-to-MSN inhibitory postsynaptic currents (IPSCs, left) were blocked by the nicotinic receptor antagonist DhβE (1 μM) and the $GABA_A$ receptor antagonist picrotoxin (0.1 mM), confirming both cholinergic and GABAergic components. n = 7 neurons from 2 mice.

**C,** CIN-to-MSN IPSC amplitudes were significantly reduced in 5xFAD mice compared to controls. $F_{(1, 39)} = 24.72$, $^{###}p < 0.001$, Two-way RM ANOVA; $^{**}p < 0.01$, $^{***}p < 0.001$ versus AD at the same stimulating intensities, Sidak's multiple comparisons post-hoc analysis. n = 20 neurons from 3 mice (Ctrl; ChAT-Cre;Ai32) and 21 neurons from 3 mice (5xFAD;ChAT-Cre;Ai32;5xFAD).

bioRxiv preprint doi: https://doi.org/10.64898/2026.01.13.699380; this version posted January 14, 2026. The copyright holder for this preprint (which was not certified by peer review) is the author/funder, who has granted bioRxiv a license to display the preprint in perpetuity. It is made available under aCC-BY 4.0 International license.

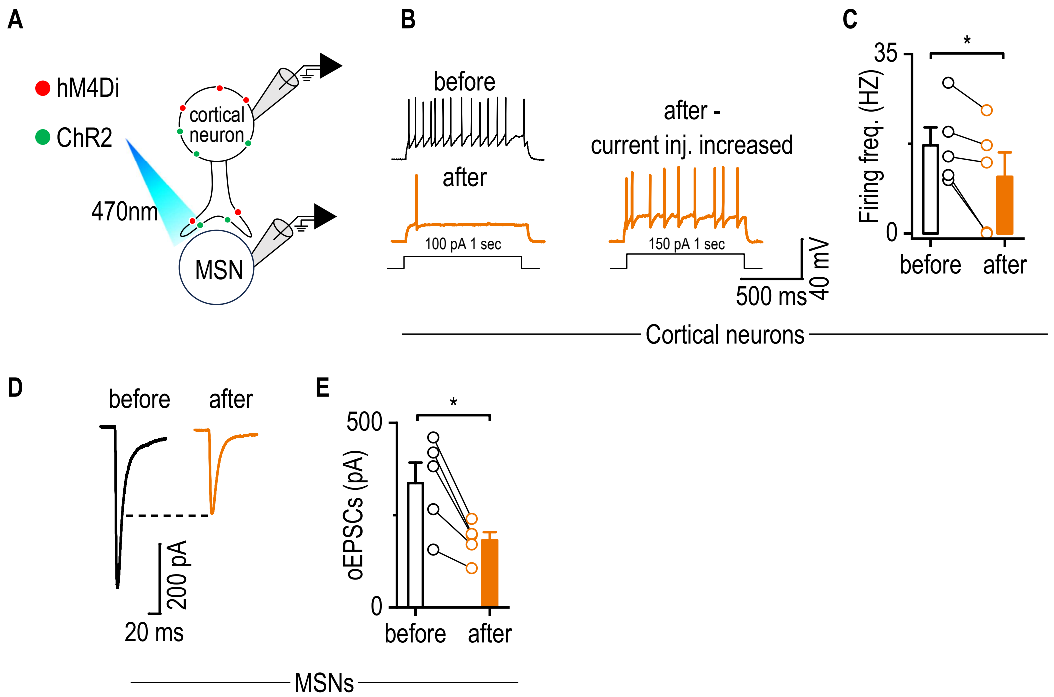


**Supplementary Figure 7. hM4Di activation reduces neuronal excitability and synaptic transmission.**

**A,** Schematic illustrating AAV infusion and patch-clamp recording strategy. AAV-hSyn-ChR2-GFP and AAV-hM4Di-mCherry were co-infused into the mPFC. Recordings were obtained from mPFC neurons co-expressing ChR2 and hM4Di, and from postsynaptic MSNs in the DMS that did not express either construct.

**B,** Sample trace showing decreased excitability of hM4Di-expressed mPFC neuron after hM4Di activation.

**C,** hM4Di activation significantly reduced excitability. Paired t-test, $^{*}p < 0.05$.

**D,** Sample trace showing decreased oEPSC amplitude after hM4Di activation.

**E,** hM4Di activation significantly reduced oEPSC amplitude. Paired t-test, $^{*}p < 0.05$.

bioRxiv preprint doi: https://doi.org/10.64898/2026.01.13.699380; this version posted January 14, 2026. The copyright holder for this preprint (which was not certified by peer review) is the author/funder, who has granted bioRxiv a license to display the preprint in perpetuity. It is made available under aCC-BY 4.0 International license.

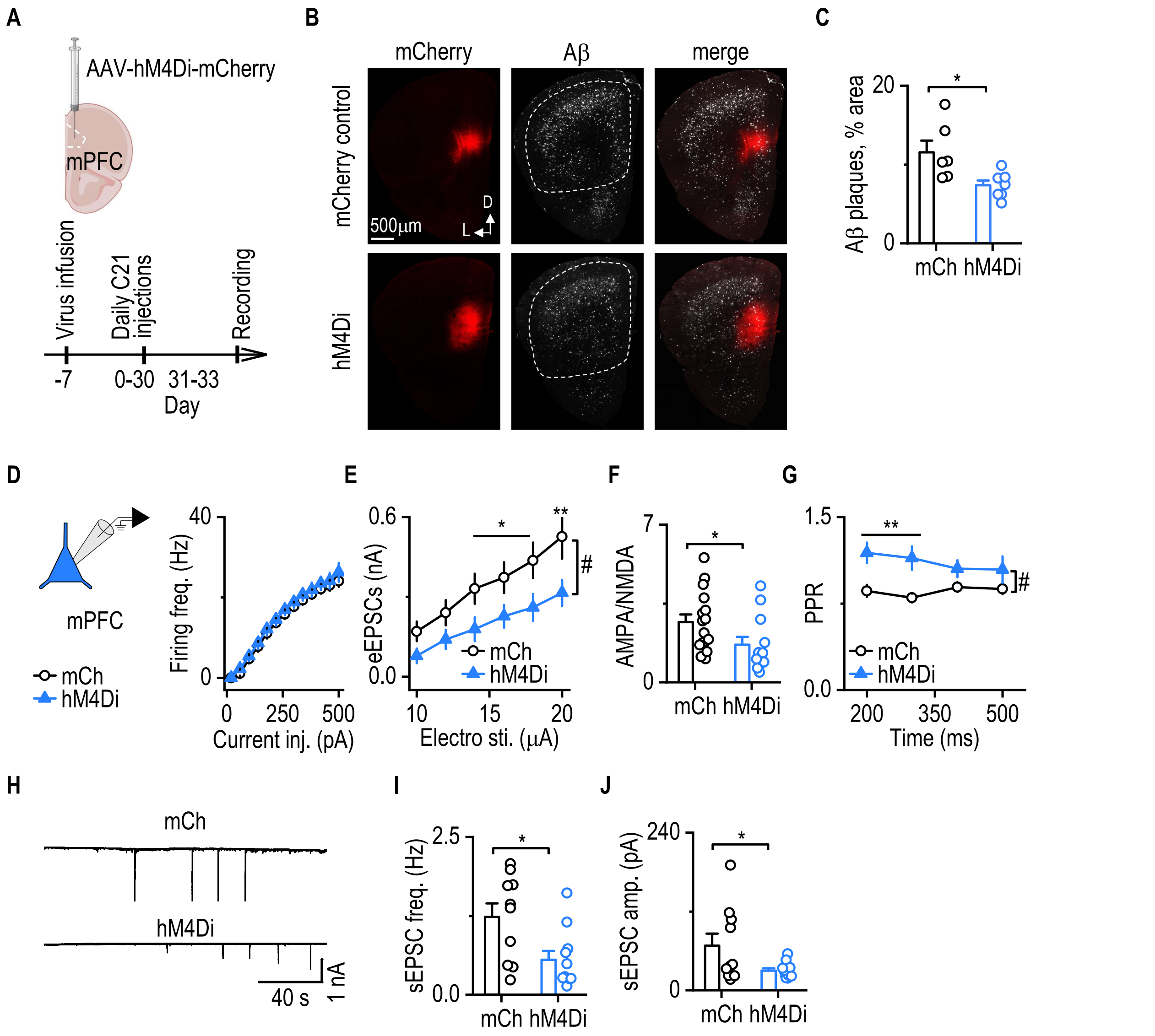


**Supplementary Figure 8. Sustained chemogenetic inhibition of cortical neurons normalizes glutamatergic transmission and results in lower Aβ accumulation in the cortex.**

**A**, Schematic illustrating the experimental timeline. At 3 months of age, 5xFAD mice were infused with either AAV-hM4Di-mCherry or AAV-mCherry in the mPFC and received daily intraperitoneal C21 injections (1 mg/kg) for four weeks. Behavioral testing was conducted before brain slices were collected for electrophysiological recordings and Aβ staining following the final C21 injection.

**B,** Representative images showing Aβ staining and quantification regions (dashed area) in

bioRxiv preprint doi: https://doi.org/10.64898/2026.01.13.699380; this version posted January 14, 2026. The copyright holder for this preprint (which was not certified by peer review) is the author/funder, who has granted bioRxiv a license to display the preprint in perpetuity. It is made available under aCC-BY 4.0 International license.

hM4Di- and mCherry-injected 5xFAD mice.

**C,** The percentage of area covered by Aβ plaques was significantly lower in hM4Di-injected mice than in mCherry-injected controls. unpaired t-test, $t_{11} = 2.751$, $p = 0.0188$. *$p < 0.05$. n = 6 mice (mCh); 7 mice (hM4Di).

**D,** Excitability of mCherry-positive mPFC neurons did not differ between hM4Di and mCherry (mCh) groups. Two-way RM ANOVA, $F_{(1,24)} = 0.068$, $p = 0.797$. n= 13 neurons from 4 mice (mCh and hM4Di).

**E,** eEPSC amplitudes in mCherry-positive mPFC neurons were significantly lower in hM4Di-injected mice than in mCherry controls. Two-way RM ANOVA, $F_{(1,32)} = 4.835$, $p = 0.035$, $^{\#}p < 0.05$; Sidak's multiple comparisons post-hoc analysis, *$p < 0.05$, **$p < 0.01$ versus mCh at the same stimulating intensities. n = 15 neurons from 4 mice (mCh) and 19 neurons from 4 mice (hM4Di).

**F,** AMPA/NMDA ratios were lower in hM4Di-injected mice than in mCherry controls. Unpaired t-test. $t_{26} = 2.048$, $p = 0.051$. *$p < 0.05$. n = 16 neurons from 4 mice (mCh) and 12 neurons from 4 mice (hM4Di).

**G,** PPRs of mCherry-positive mPFC neurons were higher in hM4Di-injected mice than in mCherry controls. Two-way RM ANOVA, $F_{(1,33)} = 7.153$, $p < 0.05$; $^{\#}p < 0.05$; Sidak's multiple comparisons post-hoc analysis, **$p < 0.01$ versus WT at the same time interval. n= 16 neurons from 4 mice (mCh) and 19 neurons from 4 mice (hM4Di).

**H,** Representative traces of sEPSCs recorded from mCherry-positive mPFC neurons in both groups.

**I and J,** Both the frequency and amplitude of sEPSCs in mPFC-mCherry positive neurons were lower for hM4Di- than mCherry-injected 5xFAD mice. unpaired t-test, $t_{19} = 2.691$, $p = 0.0145$(**G**);$t_{19} = 2.170$, $p = 0.0429$; *$p < 0.05$. n= 10 neurons from 4 mice (mCh) and 11 neurons from 4 mice (hM4Di).

bioRxiv preprint doi: https://doi.org/10.64898/2026.01.13.699380; this version posted January 14, 2026. The copyright holder for this preprint (which was not certified by peer review) is the author/funder, who has granted bioRxiv a license to display the preprint in perpetuity. It is made available under aCC-BY 4.0 International license.

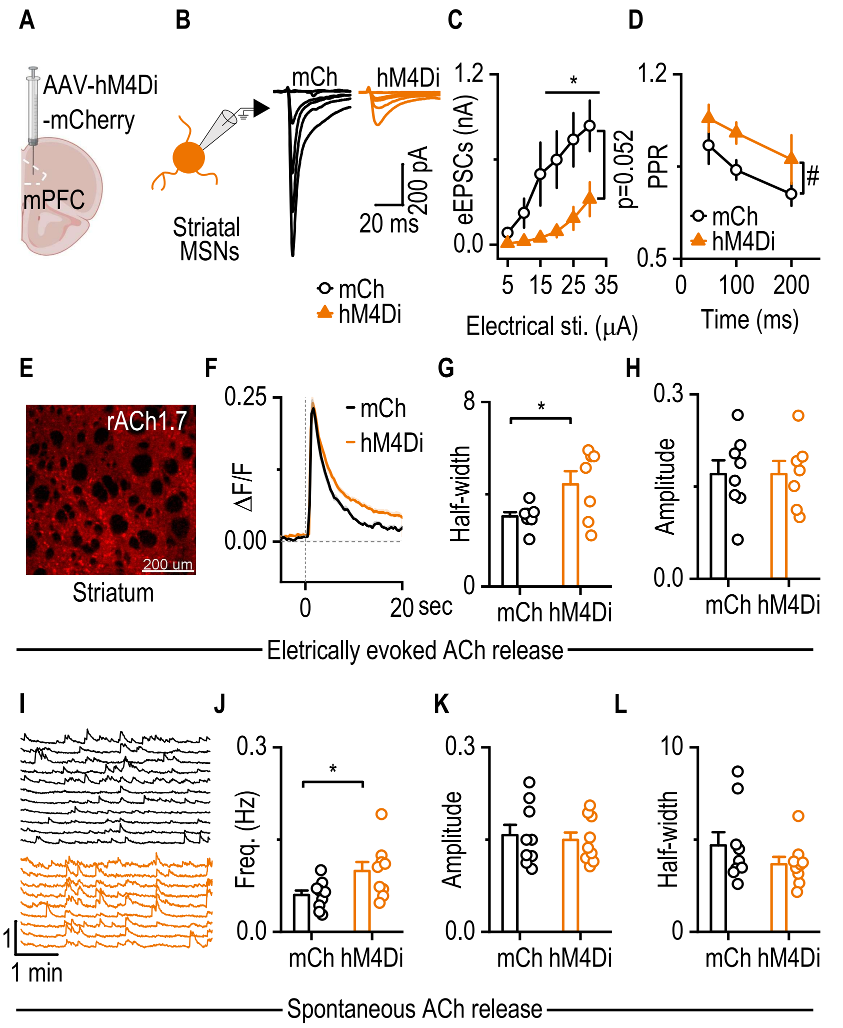


**Supplementary Figure 9. Sustained chemogenetic inhibition of cortical neurons leads to decreased glutamatergic transmission and increased striatal ACh levels.**

**A**, Schematic showing AAV-hM4Di-mCherry or AAV-mCherry infusion into the mPFC.

**B,** Representative trace of eEPSCs recorded from DMS MSNs in mCherry- and hM4Di-injected 5xFAD mice.

**C**, eEPSC amplitudes in DMS MSNs were smaller in hM4Di-injected 5xFAD mice than in mCherry-injected 5xFAD mice. Two-way RM ANOVA, $F_{(1,15)} = 4.435$, $p = 0.052$; Sidak's multiple comparisons post-hoc analysis, *$p < 0.05$ versus hM4Di at the same stimulating intensities. n = 10 neurons from 4 mice (mCh); 7 neurons from 3 mice (hM4Di).

bioRxiv preprint doi: https://doi.org/10.64898/2026.01.13.699380; this version posted January 14, 2026. The copyright holder for this preprint (which was not certified by peer review) is the author/funder, who has granted bioRxiv a license to display the preprint in perpetuity. It is made available under aCC-BY 4.0 International license.

**D**, PPR in DMS MSNs was higher in hM4Di- than in mCherry-injected 5xFAD mice. Two-way RM ANOVA, $F_{(1,17)} = 5.213$, $p = 0.036$. $^{\#}p < 0.05$. n = 11 neurons from 4 mice (mCh); 8 neurons from 3 mice (hM4Di).

**E**, Representative image showing red ACh sensor (rACh1.7) expression in striatal slices from 5xFAD mice.

**F**, Sample traces of evoked striatal ACh release recorded from rACh1.7-expressing slices.

**G and H**, The half-width (G), but not the amplitude (H), of evoked ACh release was greater in hM4Di-injected mice than in mCherry-injected 5xFAD controls. unpaired t-test, $t_{13} = -2.448$, $p < 0.05$ for half-width; Fig. 7D, $t_{13} = 0.00147$, $p > 0.05$ for amplitude. $^{*}p < 0.05$. n = 8 slices from 4 animals (G and H, mCh), 7 slices from 4 animals (G and H, hM4Di).

**I**, Sample traces of spontaneous striatal ACh release.

**J-L**, The frequency (J), but not the amplitude (K) or half-width (L), of spontaneous ACh release was significantly higher in hM4Di-injected 5xFAD mice compared to mCherry-injected 5xFAD mice. unpaired t-test, (J), $t_{16} = 2.377$, $p = 0.0303$ for frequency; (K) $t_{16} = 0.3087$, $p > 0.05$ for amplitude; (L) $U = 29$, $p > 0.05$ for half-width. $^{*}p < 0.05$. n = 9 slices from 4 animals (mCh and hM4Di).

bioRxiv preprint doi: https://doi.org/10.64898/2026.01.13.699380; this version posted January 14, 2026. The copyright holder for this preprint (which was not certified by peer review) is the author/funder, who has granted bioRxiv a license to display the preprint in perpetuity. It is made available under aCC-BY 4.0 International license.

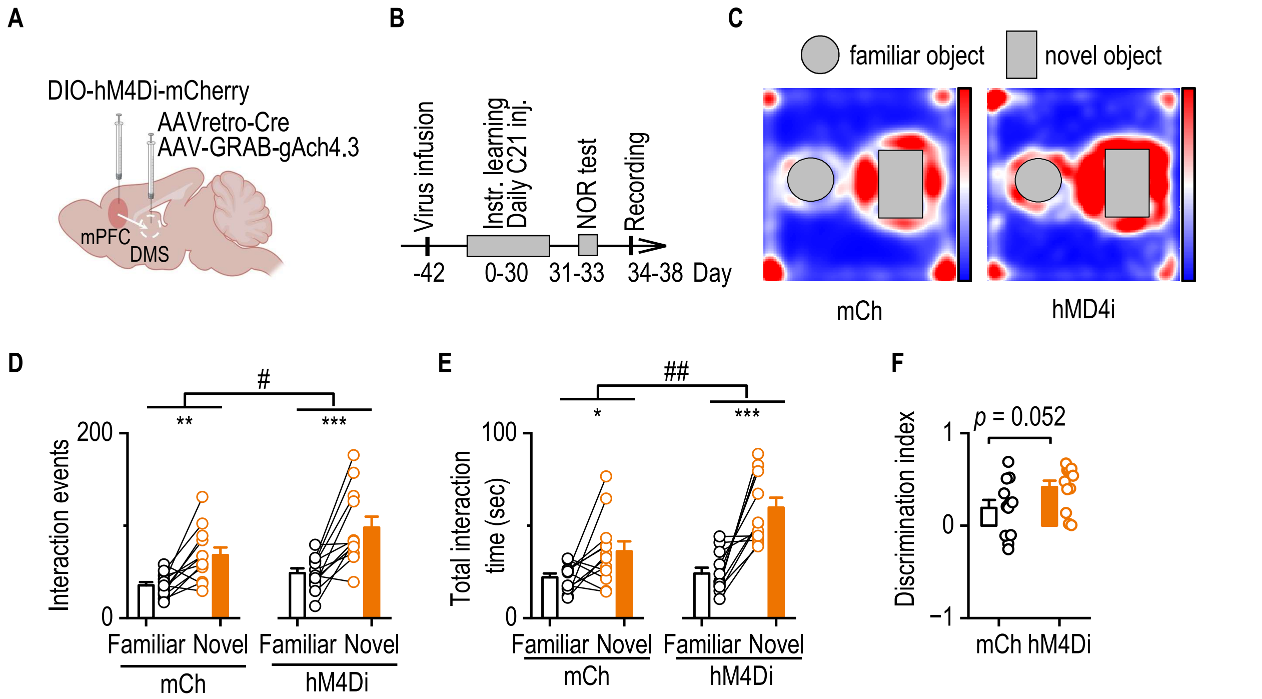


**Supplementary Figure 10. Sustained inhibition of mPFC to DMS circuit improved cognitive function in 5xFAD mice.**

**A,** Schematic illustrating viral infusion strategy in 5xFAD mice. AAV-retro-Cre and AAV-GRAB-ACh4m were infused into the DMS, and AAV-DIO-hM4Di-mCherry or AAV-DIO-mCherry into the mPFC.

**B,** Experimental timeline. The novel object recognition (NOR) test was performed after one month of sustained chemogenetic inhibition.

**C**, Heatmaps showing time spent interacting with novel and familiar objects during the 5-minute testing session.

**D**, Both hM4Di- and mCherry-injected 5xFAD mice showed more interaction events with the novel object than the familiar object. However, hM4Di-injected mice exhibited significantly more novel object interactions than mCherry controls. Two-way RM ANOVA, $F_{(1,22)} = 6.246$, $p = 0.020$, $^{\#}p < 0.05$; Sidak's multiple comparisons post-hoc analysis, $^{*}p < 0.05$. n = 12 mice (mCh and hM4Di).

bioRxiv preprint doi: https://doi.org/10.64898/2026.01.13.699380; this version posted January 14, 2026. The copyright holder for this preprint (which was not certified by peer review) is the author/funder, who has granted bioRxiv a license to display the preprint in perpetuity. It is made available under aCC-BY 4.0 International license.

**E**, Both groups spent more total time interacting with the novel object than the familiar object, with hM4Di-injected mice exhibiting significantly longer interaction times overall. Two-way RM ANOVA, $F_{(1,22)} = 10.753$, $p = 0.003$, $^{\#\#}p < 0.01$; Sidak's multiple comparisons post-hoc analysis, $^{*}p < 0.05$. n = 12 mice (mCh and hM4Di).

**F**, hM4Di-injected 5xFAD mice showed a higher discrimination index than mCherry-injected 5xFAD mice. unpaired t-test, $t_{22} = -2.055$, $p = 0.0519$. n = 12 mice (mCh and hM4Di).

bioRxiv preprint doi: https://doi.org/10.64898/2026.01.13.699380; this version posted January 14, 2026. The copyright holder for this preprint (which was not certified by peer review) is the author/funder, who has granted bioRxiv a license to display the preprint in perpetuity. It is made available under aCC-BY 4.0 International license.

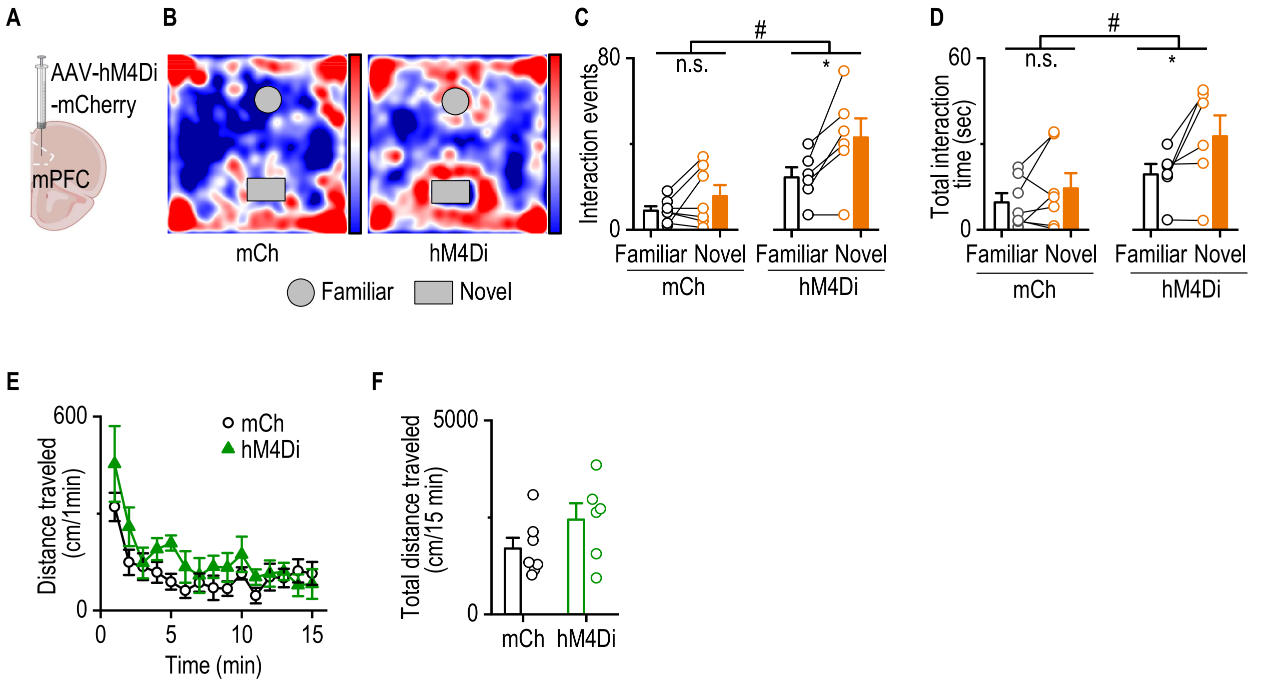


**Supplementary Figure 11. Sustained inhibition of mPFC neurons improves cognitive function in 5xFAD mice without impairing locomotor activity.**

**A**, Schematic showing AAV-hM4Di-mCherry or AAV-mCherry infusion in mPFC.

**B**, Heatmaps of animals that interacted with novel and familiar objects.

**C**, hM4Di-injected 5xFAD mice exhibited more interaction events with the novel object than controls in a 5-min session. Two-way RM ANOVA, $F_{(1,11)} = 9.460$, $p = 0.011$, $^{\#}p < 0.05$; Sidak's multiple comparisons post-hoc analysis, $^{*}p < 0.05$. n = 7 mice (mCh); 6 mice (hM4Di).

**D**, hM4Di-injected 5xFAD mice exhibited longer total interaction time with the novel object than controls in a 5-min session. Two-way RM ANOVA, $F_{(1,11)} = 4.906$, $p = 0.049$, $^{\#}p < 0.05$; Sidak's multiple comparisons post-hoc analysis, $^{*}p < 0.05$. n = 7 mice (mCh); 6 mice (hM4Di).

**E, F,** hM4Di-injected and mCherry-injected 5xFAD mice exhibited similar spontaneous locomotor activity as measured by time-course (E, Two-way RM ANOVA, $F_{(1,11)} = 2.267$, $p > 0.05$) and total distance traveled (F, unpaired t-test, $t_{11} = -1.506$, $p > 0.05$). n = 7 (E and F, mCh), 6 (E and F, hM4Di).
